# SPOCK2 gene expression is downregulated in pancreatic ductal adenocarcinoma cells and correlates with prognosis of patients with pancreatic cancer

**DOI:** 10.1007/s00432-023-04845-5

**Published:** 2023-05-15

**Authors:** Ughur Aghamaliyev, Kaifeng Su, Maximilian Weniger, Dominik Koch, Jan G. D‘Haese, Jens Werner, Alexandr V. Bazhin

**Affiliations:** 1 Department of General, Visceral, and Transplant Surgery, Hospital of the University of Munich, Ludwig-Maximilians-University Munich, Marchioninistr. 15, 81377 Munich, Germany; 2 German Cancer Consortium (DKTK), Partner Site Munich and German Cancer Research Center (DKFZ), Heidelberg, Germany; 3 Bavarian Cancer Research Center (BZKF), Erlangen, Germany

**Keywords:** SPOCK2, SPARC, ECM, PDAC, Pancreatic cancer, Hypermethylation

## Abstract

**Objectives:**

Pancreatic ductal adenocarcinoma (PDAC) represents a widespread form of malignant pancreatic neoplasms and a leading oncologic cause of death in Europe and the USA. Despite advances in understanding its molecular biology, the 5-year survival rate remains low at 10%. The extracellular matrix in PDAC contains proteins, including SPOCK2, which are essential for tumorigenicity and drug resistance. The present study aims to explore the possible role of SPOCK2 in the pathogenesis of PDAC.

**Materials and methods:**

Expression of SPOCK2 was evaluated in 7 PDAC cell lines and 1 normal pancreatic cell line using quantitative RT-PCR. Demethylation of the gene was carried out using 5-aza-2'-deoxycytidine (5-aza-dC) treatment with subsequent validation Western Blot analysis. In vitro downregulation of SPOCK2 gene was performed using siRNA transfection. MTT and transwell assays were employed to evaluate the impact of the SPOK2 demethylation on the proliferation and migration of PDAC cells. KM Plotter was applied to analyze a correlation between SPOCK2 mRNA expression and the survival of PDAC patients.

**Results:**

In contrast to the normal pancreatic cell line, SPOCK2 expression was significantly downregulated in PDAC cell lines. Treatment with 5-aza-dC, led to increase in SPOCK2 expression in the cell lines tested. Importantly, compared with control cells, transfected with SPOCK2 siRNA cells exhibited increased growth rates and more migration ability. Finally, we demonstrated that a high SPOCK2 expression level correlated with longer overall survival of patients with PDAC.

**Conclusion:**

The expression of SPOCK2 is downregulated in PDAC as a result of hypermethylation of its corresponding gene. SPOCK2 expression as well as the demethylation of its gene could be a potential marker for PDAC.

## Introduction

Pancreatic ductal adenocarcinoma (PDAC) represents the most prevalent variant of malignant pancreatic neoplasms and the fourth leading oncological cause of death in Europe and the USA (Siegel et al. 2022). This dismal prognosis of PDAC is due to the distant metastases and locally advanced tumors at the time of diagnosis (Isaji et al. 2018). Despite significant progress in the understanding of the molecular biology underlying PDAC, the five-year survival rate for patients with this malignancy remains at approximately 10% (Park et al. 2021). Findings suggest that not only cancer cells, but also desmoplastic reaction contribute to chemoresistance in PDAC (Aghamaliyev et al. 2016). Tumor stroma is composed of cellular components such as fibroblasts, stellate, immune and nerve cells, and acellular components. Together, cellular and acellular components form the extracellular matrix (ECM) (Whatcott et al. 2015). The ECM in PDAC contains collagens, integrins, proteoglycans, and glycoproteins. Of note, these ECM components are essential for tumorigenicity and drug resistance in PDAC (Karamitopoulou 2019).

SPOCK2 (SPARC (osteonectin), cwcv and kazal-like domains proteoglycan 2), also identified as testican-2, is a proteoglycan that belongs to the SPARC family and is present in the extracellular matrix (Nordgard et al. 2008). As SPOCK2 was reported to undergo hypermethylation in some cancer entities in 2008 (Chung et al. 2008), it has gained attention in this field. For instance, SPOCK2 has been reported to contribute to endometrium cancer progression, in vitro (Ren et al. 2020). In prostate cancer, SPOCK2 mRNA level was lower in contrast to benign prostate hyperplasia, and the upregulation of SPOCK2 in prostate cancer cell lines via transfection inhibited cell invasion and migration, in vitro (Liu et al. 2019). Using TCGA and CPTAC databases, SPOCK2 was reported to be downregulated in lung adenocarcinoma (LUAD) in comparison to normal lung tissue (J. Zhao et al. 2020). Moreover, the recent in silico study revealed a significant association between high mRNA expression of SPOCK2 and extended OS in patients diagnosed with LUAD (J. Zhao et al. 2020). Among gastrointestinal malignancies, SPOCK2 expression was reported in colon cancer (Sambuudash et al. 2017). Nonetheless, the existing literature lacks investigations concerning the biological mechanisms and prognostic significance of SPOCK2 in CRC. Indeed, SPOCK2 was reported to be correlated with immune infiltration in PDAC using bioinformatics tools (Lu et al. 2021), but its role in PDAC progression has not been yet researched.

The main aim of this study was to become the first hint of the potential involvement of SPOCK2 in the pathogenesis of PDAC.

## Materials and methods

### Materials

The acquisition and preservation of the seven PDAC cell lines, Panc1, Dang, Aspc1, Capan1, Capan2, Miapaca-2, and Bxpc3, were carried out through purchase from ATCC and subsequent storage in bio-liquid nitrogen tanks. The normal human pancreatic duct epithelial (HPDE) cell line, on the other hand, was obtained from The Technical University of Munich's laboratory. All reagents and kits for total RNA extraction were from Qiagen, Inc. (Qiagen, Hilden, Germany). For cDNA synthesis, SuperScript IV VILO Master Mix was obtained from Thermo Fisher, Inc. (Schwerte, Germany). Equipment for RT-PCR was BioRad CFX96 RealTime PCR from BioRad Laboratories, Inc. (California, USA). 5 mg of 5-aza-dC lyophilized powder in a glass insert was from Sigma-Aldrich (Darmstadt, Germany). For siRNA silencing, Lipofectamine^™^ RNAiMAX Transfection Reagent was purchased from Invitrogen, (California, USA). Control (non-sil.) siRNA was from Qiagen (Hilden, Germany). The anti-SPOCK2 polyclonal antibody (1:1000) was purchased from Abcam (Cambridge, UK), and GAPDH (1:5000) and ZO-1 (1:1000) from Cell Signaling Technology (Frankfurt, Germany). The BCA protein Assay kit was procured from Thermo Fisher Scientific (Schwerte, Germany).

### Cell culture

The PDAC cells of Panc1 and Miapaca-2 cell lines were cultivated using Dulbecco’s modified Eagle’s medium supplemented with 10% (v/v) fetal bovine serum. The five PDAC cell lines Dang, Aspc1, Capan1, Capan2, and Bxpc3 were cultured in RPMI 1640 medium supplemented with 10% fetal bovine serum. The HPDE cell line was cultured in Keratinocyte-SFM (Serum-Free Medium), supplemented with 10% FBS, 2.5 μg EGF (epidermal growth factor), and 25 mg BPE (bovine pituitary extract). The cell lines were cultivated under standard conditions with 5% CO2 at 37 °C, were subjected to mycoplasma screening every four months in compliance with laboratory protocols, and were annually validated by IDEX BioResearch (Ludwigsburg, Germany).

### siRNA silencing

RNA interference was performed according to the previous description (M. Zhao et al. 2008). In brief, the Capan2 cells were transfected 24 h post-culture with SPOCK2 small interfering RNA (siRNA) following the manufacturer’s instructions. Capan2 cells transfected with a non-target control siRNA were used as controls.

### Treatment with 5-aza-2’-deoxycytidine (5-aza-dC)

The PDAC cells were cultivated in a 6-well plate overnight at 37 °C in a 5% CO2 incubator. After 24 h, cells were treated with 5-aza-dC a concentration of 1 μM for 48 h. Following treatment, RNA extraction from the cells was immediately carried out.

### RNA isolation and quantitative RT‑PCR

Total RNA was extracted using Qiagen RNeasy Micro Kit according to the manufacturer’s protocol. For cDNA synthesis, SuperScript IV VILO Master Mix was applied. Gene expression was quantified using QuantiNovaTM SYBR Green PCR Kit and performed in a BioRad CFX96 RealTime PCR equipment. Standardization of cDNA variation was achieved using the housekeeping genes, 18S, B2M, and GAPDH. Each group was subjected to a minimum of three distinct experiments. 2^−ΔCT^ (ΔCT = CT_target gene_ – CT_housekeeping gene_) was used to determine the relative gene expression level.

### Western blot analysis

Cells were lysed using RIPA buffer at 4 °C for 40 min, and cell lysates were harvested by centrifugation at 10,000 rpm for 10 min to obtain the supernatants. Soluble cell lysate fractions were quantitated using a Micro BCA protein assay kit. Proteins were transferred onto polyvinylidene fluoride membranes and blocked with 5% bovine serum albumin (BSA) for 1 h at room temperature. The membrane was incubated with the following primary antibodies: SPOCK2 Antibody (dilution: 1:1000), ZO-1 (dilution: 1:1000), and GAPDH (dilution 1:5000) at 4 °C overnight. Ultimately, immunoreactive bands were assessed in the darkroom using autoradiography film. Image J software was utilized to determine the grey values of the bands, which were subsequently subjected to statistical analysis.

### MTT assay

Assessment of cell viability was performed through the implementation of the 3-(4,5-Dimethylthiazol-2-yl)-2,5-Diphenyltetrazolium Bromide assay. Following transfection for 24 h, 5 × 10^3^ cells were seeded in each well of a 96-well plate. Before counting, cells were mixed with a fresh medium containing 5 mg/ml MTT the cells were incubated at 37 °C, for an additional 4 h. Absorbance measurements of each well were carried out at 570 nm, with a background wavelength of 670 nm, utilizing a VersaMax microplate reader. Wells without cells served as controls. The test was independently repeated at least three times.

### Transwell assay

Transwell assay was conducted for analysis of cell migration. For this purpose, 100,000 transfected cells or control cells, in serum-free medium, were planted into the upper chamber of a Transwell insert (8-mm pore size). The lower chamber was supplemented with 600 μl medium containing 20% FBS. The lower chamber was supplemented with 600 μl medium containing 20% FBS. Following incubation at 37 °C and 5% CO2 for 36 h, the Transwell chamber was extracted, and the medium in the well was eliminated and washed with calcium-free PBS. The cells were then fixed with 4% paraformaldehyde for 30 min and subsequently stained with 0.1% Crystal Violet (CV) for additional 30 min at room temperature. The upper non-migrated cells were cautiously wiped off with a cotton swab and counted under a microscope.

### Flow cytometry analysis

Subsequent to transfection for a duration of 24 h, a quantity of 1X10^5^ cells was seeded in each well of a 6-well plate and cultured with complete medium for 48 h. The cells were subsequently collected and treated with 20 μl bromodeoxyuridine (BrdU) solution (1 mM BrdU in 1xPBS) per well, followed by a 1-h incubation (37 °C, 5% CO2). After centrifugation and discarding the supernatant, the cells were fixed and permeabilized with 100 µl BD Cytofix/Cytoperm Buffer. Subsequently, the cells were incubated with 100 µl of BD Cytoperm Plus Buffer and re-fixated with 100 µl BD Cytofix/Cytoperm Buffer, as mentioned above. The cells were then resuspended with 100 μl diluted DNase (300 μg/ml) and incubated at 37 °C for a duration of 1 h. As a next step, the cells were resuspended with 50 µl of BD Perm/Wash Buffer containing diluted fluorescent anti-BrdU and incubated for 20 min at room temperature. Finally, the stained cells were immediately measured with the cytometer (FACS Fortessa) after adding 1 ml of staining buffer to each tube.

### Bioinformatic analysis

The cBioPortal (http://www.cbioportal.org/), an open-source platform for cancer genomics, enables the exploration of multidimensional datasets of cancer genomes. To study SPOCK2 in PDAC, the Firehose Legacy dataset, consisting of data from 185 PDAC patients with pathological results, was selected for analysis using the cBioPortal tool. The genomic profiles of PDAC patients, including the frequency of gene mutations and mRNA expression levels of SPOCK2 (as measured by RNA Seq V2 RSEM), were analyzed using the online tool provided by the cBioPortal (Cerami et al. 2012). KM Plotter (https://kmplot.com) bioinformatics tool was employed to assess the prognostic significance of SPOCK2 (Nagy et al. 2018). The ICGC-PACA-CA cohort was sourced from the ICGC database (https://dcc.icgc.org/) (International Cancer Genome et al. 2010). After removing duplicate and incomplete data, a total of 167 PDAC patients with comprehensive clinical information were selected for analysis. Kaplan–Meier analysis was employed to assess the variance in OS rates among the aforementioned patient cohort.

### Statistical analysis

All experiments were conducted at least in triplicate, with each repetition performed independently. The statistical analysis and graphical representations were carried out using GraphPad Prism, SPSS, and R software. The data were expressed as mean ± SEM. The student *t* test was employed to assess the differences between the groups, whereby a p-value of less than 0.05 was deemed to be statistically significant. The correlation between two continuous data sets was evaluated utilizing Pearson’s test and was presented as *p* and *r* values, where a value of *r* > 0.3 and *p* < 0.05 was considered statistically significant.

## Results

### SPOCK2 gene expression is downregulated in PDAC cells

**Fig. 1:**
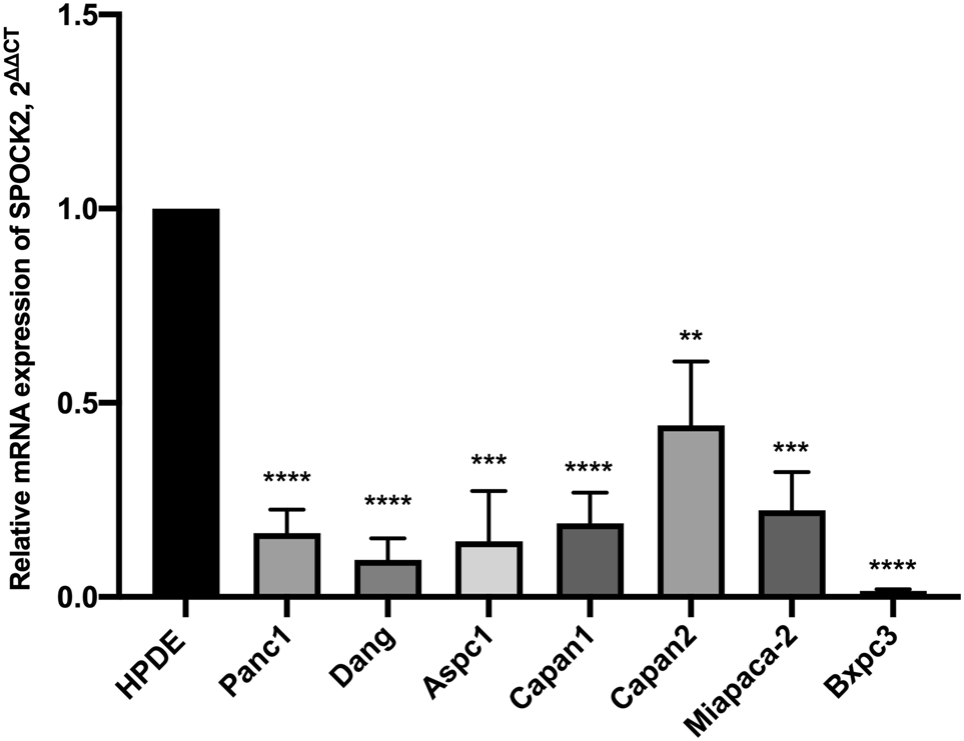
The expression of SPOCK2 in the normal pancreatic cell line and seven pancreatic cancer cell lines. The mean expression levels of housekeeping genes RPS18 and B2M were employed as a reference and measured using the 2^−ΔCT^ method. Each of the pancreatic cancer cell lines was evaluated individually in comparison to HPDE, with each sample tested independently at least three times. *P < 0.05, **P < 0.01, ***P < 0.001, ****P < 0.0001. The value was shown as mean ± SEM

SPOCK2 gene expression was analyzed by RT-PCR in seven established PDAC cell lines and one immortalized epithelial cell line originated from normal human pancreatic duct epithelial (HPDE) cells. The mRNA level of SPOCK2 was significantly lower in all PDAC cell lines when compared to HPDE cells **(**Fig. 1**)**.

### Treatment of PDAC cells with 5-aza-dC induces SPOCK2 gene expression

**Fig. 2:**
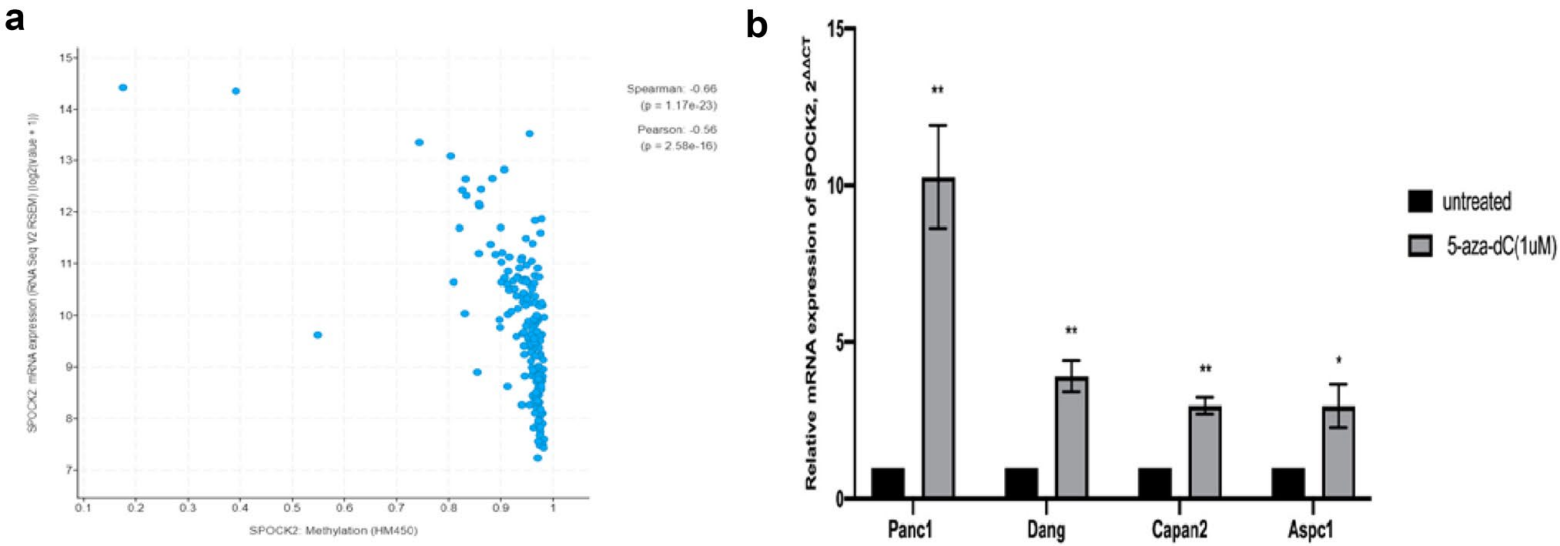
Hypermethylation of SPOCK2 in PDAC. **a** The association between gene expression and methylation of SPOCK2. The y-axis indicates the mRNA expression of SPOCK2 gene, while the x-axis represents its promoter methylation in beta values. **b** The mRNA expression of SPOCK2 in four PDAC cell lines (Panc1, Dang, Capan2, und ASPC1) following exposure to 5-aza-dC. The housekeeper gene GAPDH was utilized as a reference. The 2^−ΔCT^ method was used to quantify gene levels and normalize relative expression, with untreated groups defined as 1.0. The significance level was denoted by asterisks (*P < 0.05, **P < 0.01), and the results were presented as mean ± SEM

**Fig. 3:**
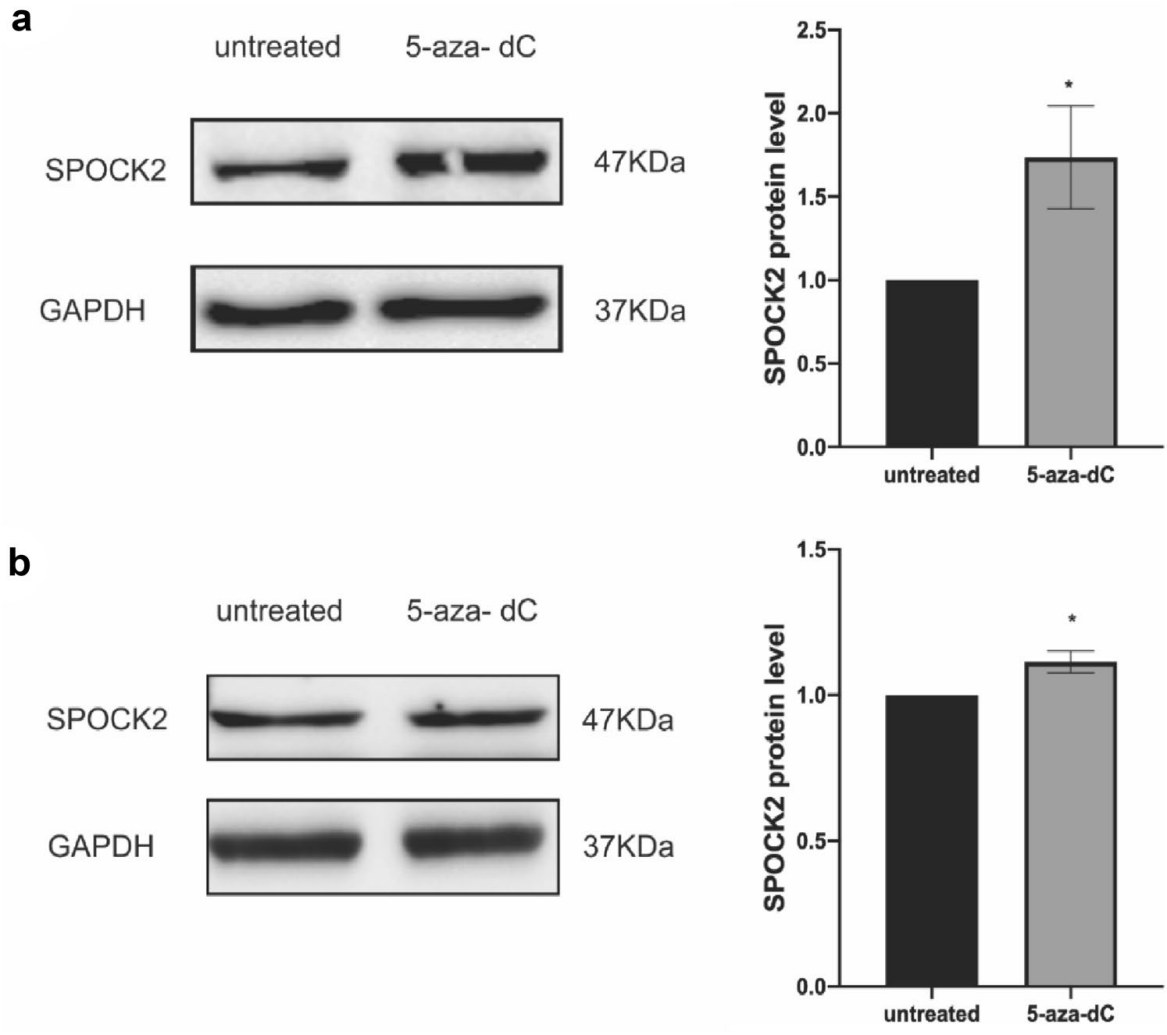
The protein expression of SPOCK2 in Panc1 and Capan2 cell lines following treatment with 5-aza-dC. Western blotting demonstrated a statistically significant increase in the expression of SPOCK2 protein in Panc1 **a** and Capan2 **b** cell lines upon exposure to 5-aza-dC. GAPDH was used as a loading control for normalization. The quantification of protein expression was performed using *ImageJ* software and represented as a bar graph. The statistical significance was denoted by *P < 0.05, and the data were presented as mean ± SEM

Taking into account that hypermethylation is one of the crucial mechanisms for the regulation of gene expression, the cbioportal tool was utilized to conduct an analysis of the mRNA expression and methylation status of SPOCK2 in PDAC. Based on the analysis, SPOCK2 methylation level was negatively correlated with its gene expression (*R* = − 0.66, *P*-value: < 0.0001) **(**Fig. 2a**)**. Of note, the beta value ranged mostly from 0.9 to 1, as a beta value exceeding 0.7 indicates strong hypermethylation. In order to further explore the possible role of hypermethylation in the downregulation of SPOCK2, four established PDAC cell lines (Aspc1, Dang, Panc1, and Capan2) were treated with 5-aza-DC. Indeed, exposure of PDAC cells to 5-aza-DC resulted in upregulation of SPOCK2 mRNA expression (*P* < 0.01 Panc1, Dang, and Capan2, *P* = 0.0468 for Aspc1) (Fig. 2b). Since Panc1 and Capan2 showed a statistically significant upregulation after 5-aza-DC exposure, we performed Western Blot analysis in protein lysates of these two PDAC cell lines (Fig. 3a,b). Of note, the upregulation of SPOCK2 expression could also be confirmed on the protein level (*P* < 0.05).

### siRNA-mediated gene silencing of SPOCK2 expression stimulates the proliferation rate and migration ability of PDAC cells

**Fig. 4:**
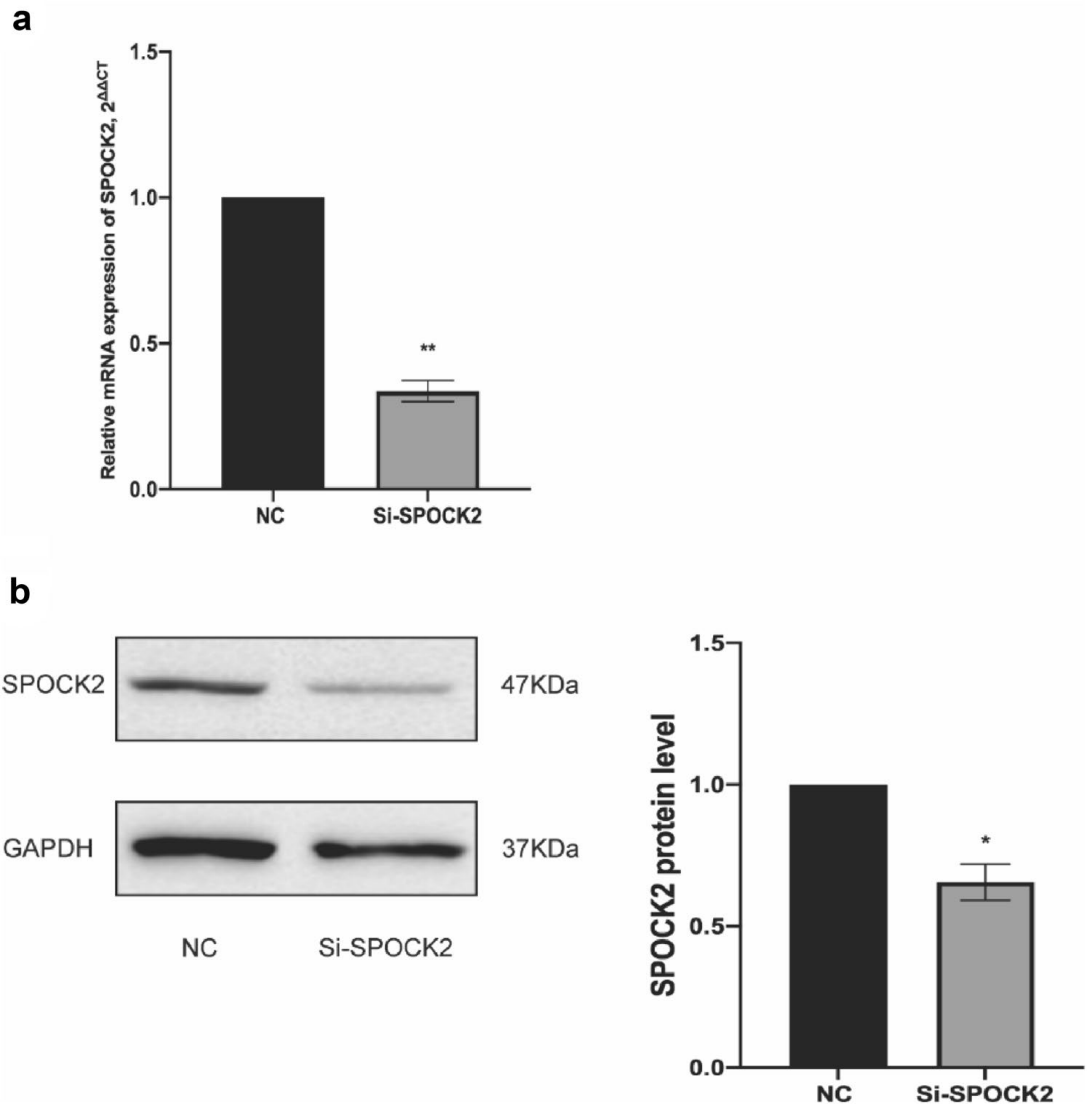
siRNA-induced changes in SPOCK2 expression levels in Capan2. **a** Gene expression levels were evaluated using qRT-PCR with the housekeeping gene GAPDH as a reference. The 2^−ΔCT^ method was used to measure gene expression and normalize the relative expression to the corresponding NC group, which was defined as 1.0. Statistical significance was determined as *P < 0.05. **b** Protein expression levels were analyzed via Western blot, with GAPDH serving as the loading control. *ImageJ* was utilized to quantify protein levels and visualize the data as a bar graph. Statistical significance was determined as *P < 0.05. Mean values were reported as ± SEM

**Fig. 5:**
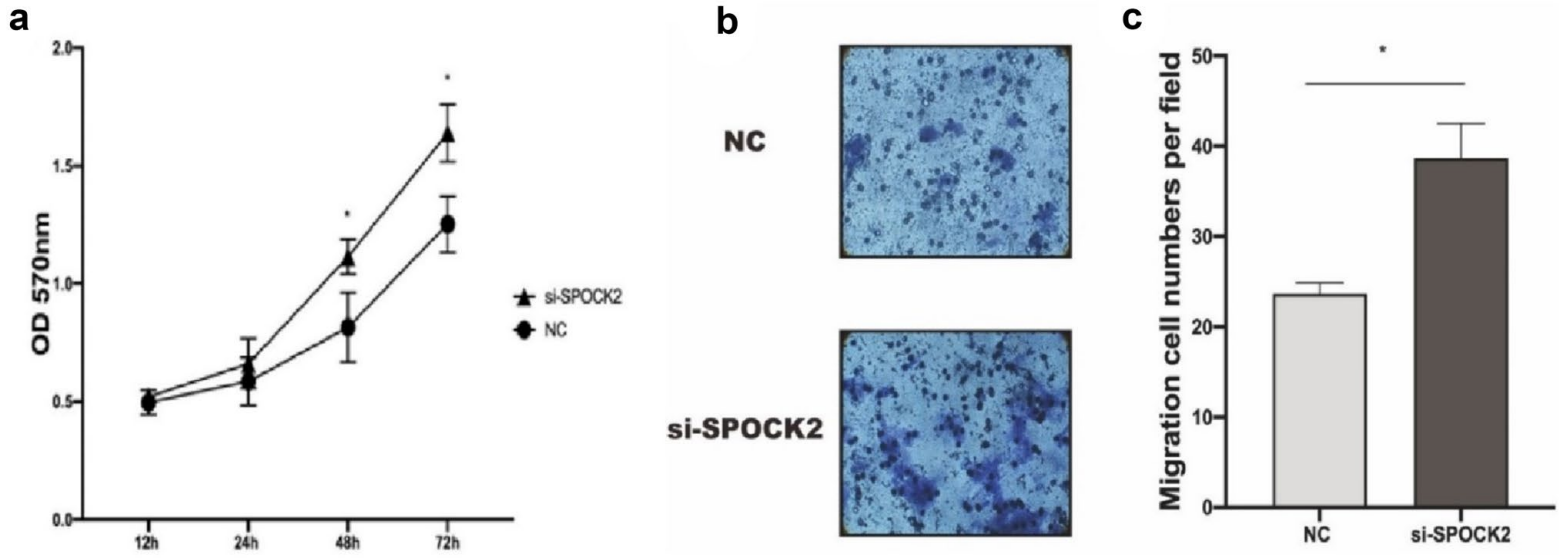
The impact of SPOCK2 knockdown on cell proliferation rate and migration ability of Capan2 cells. **a** In vitro knockdown of SPOCK2 using siRNA led to an increase in cell proliferation. **b** Representative images of the transwell assay. **c** Quantitative analysis of the transwell experiment. Statistical significance was determined as *P < 0.05, and non-significant differences were denoted as *ns*. *NC* Negative control; *si-SPOCK2* small interfering RNA for SPOCK2. Mean values were reported ± SEM

**Fig. 6:**
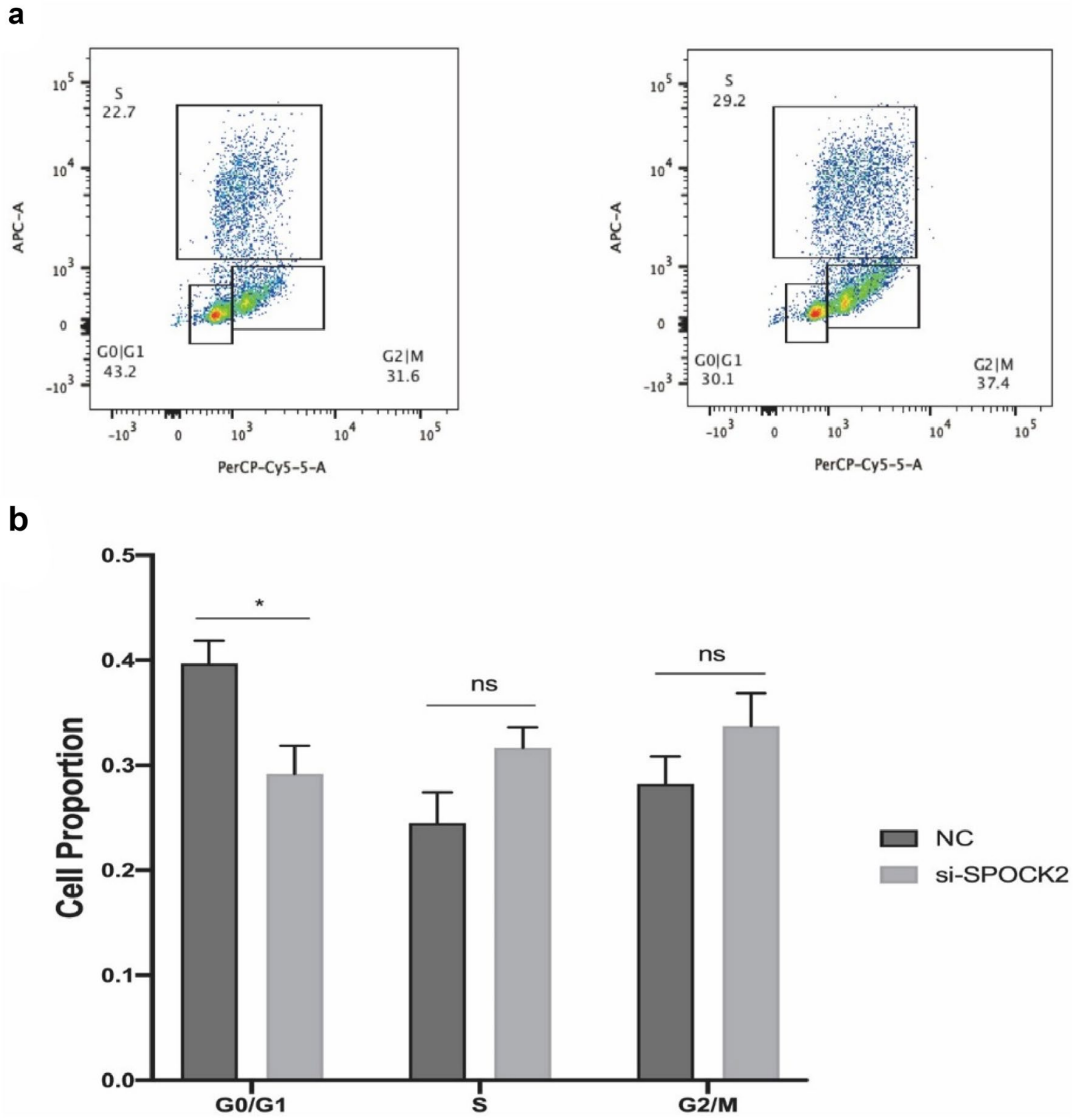
The effects of SPOCK2 knockdown on the cell cycle of Capan2 cells. **a** Changes in the cell cycle were analyzed using flow cytometry, and the results were presented as dot plots. **b** The percentage of cells in different cell cycle phases was quantified and subjected to statistical analysis. *P < 0.05, *ns* = of no significance. *NC*, negative control. *si-SPOCK2*, small interfering RNA for SPOCK2. Values were shown as mean ± SEM

To examine the role of SPOCK2 in PDAC tumorigenesis, we utilized siRNA to reduce SPOCK2 expression in Capan2 cells. Subsequently, we assessed the gene and protein expression of SPOCK2 using RT-PCR and Western blotting, respectively. The transfection of cells resulted in a significant decrease in both SPOCK2 mRNA and protein expression (*P* < 0.01; *P* < 0.05) (Fig. 4a, b**)**. Using MTT assay, we found that SPOCK2 knockdown resulted in an increase in the proportion of active cell proliferation compared with Capan2 cells transfected with nonsense siRNA as a negative control (Fig. 5a). Furthermore, we conducted flow cytometry analysis to determine whether the observed increase in cell proliferation following the suppression of SPOCK2 expression in Capan2 cells was due to alterations in the cell cycle. Cell cycle analysis showed that SPOCK2 downregulation led to G1 phase arrest in Capan2 cells (*P* < 0.05) **(**Fig. 6a, b**)**. In addition, we evaluated the migratory capacity of transfected Capan2 cells through the use of a transwell migration assay. In comparison to control cells, the migration ability of Capan2 cells with SPOCK2 siRNA silencing was significantly increased (*P* < 0.05) **(**Fig. 5b, c**)**.

### Knockdown of SPOCK2 leads to downregulation of ZO-1

**Fig. 7:**
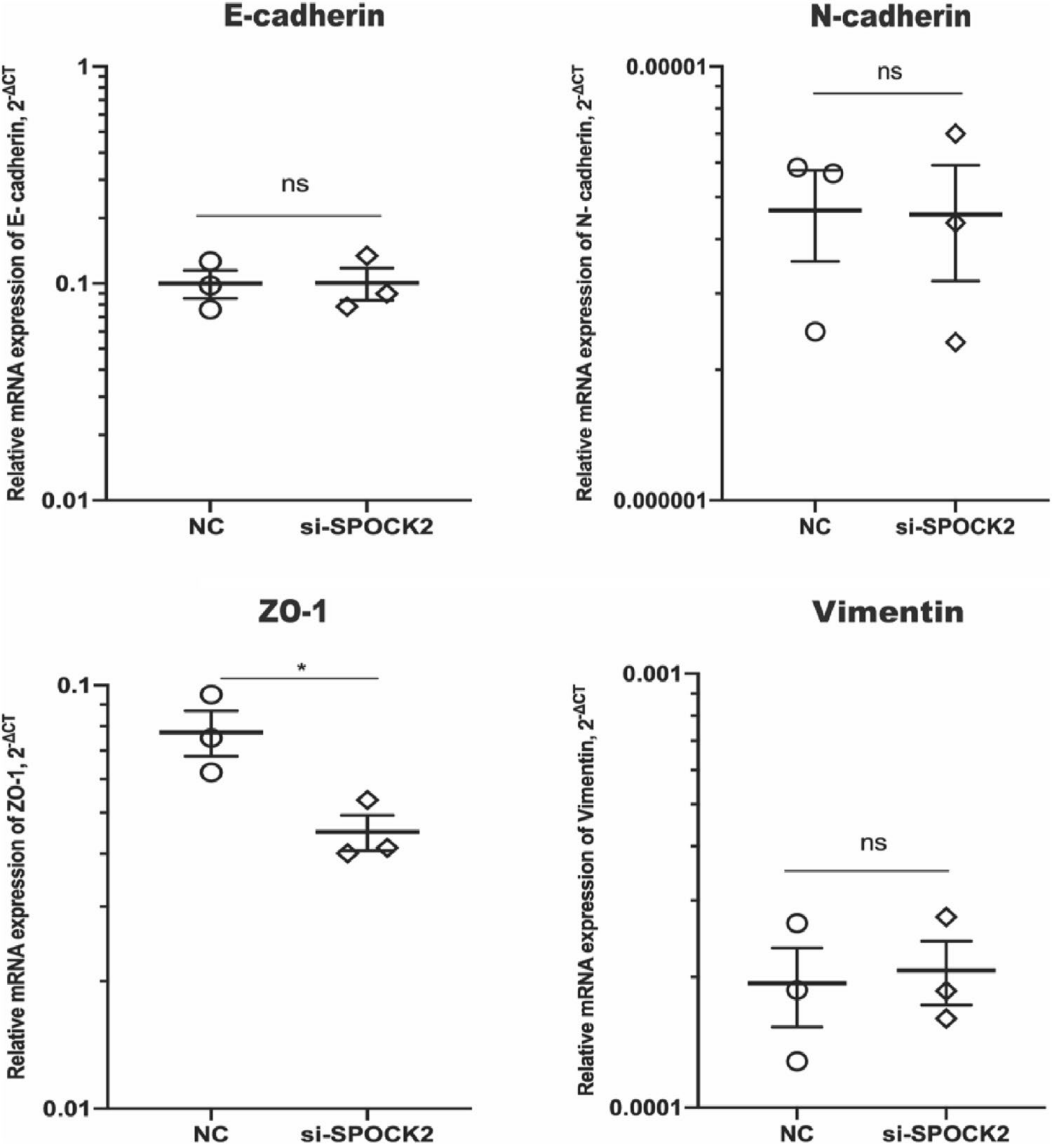
The alterations in EMT markers (E-cadherin, N-cadherin, ZO-1, Vimentin) following SPOCK2 knockdown in Capan2 cells. The gene expression levels were quantified using the 2^−ΔCT^ method with GAPDH as the reference gene. *P < 0.05. *ns* = no significance. *NC* negative control. *si-SPOCK2*, small interfering RNA for SPOCK2. Values were shown as mean ± SEM

Considering the significant role of epithelial mesenchymal transition (EMT) in cellular migration, we proceeded to explore the potential relationship between SPOCK2 and EMT markers in PDAC cells. By conducting RT-PCR, we found that gene expression of ZO-1, an epithelial marker, was significantly downregulated after SPOCK2 silencing (*P* < 0.05) (Fig. 6). ZO-1 downregulation could be validated at protein level by Western blot analysis, as well (Fig. 7). However, we did observe no difference in gene expression of E-cadherin, N-cadherin, and Vimentin after cell transfection with SPOCK2 siRNA.

### High SPOCK2 mRNA levels correlate with better overall survival in patients with PDAC

**Fig. 8:**
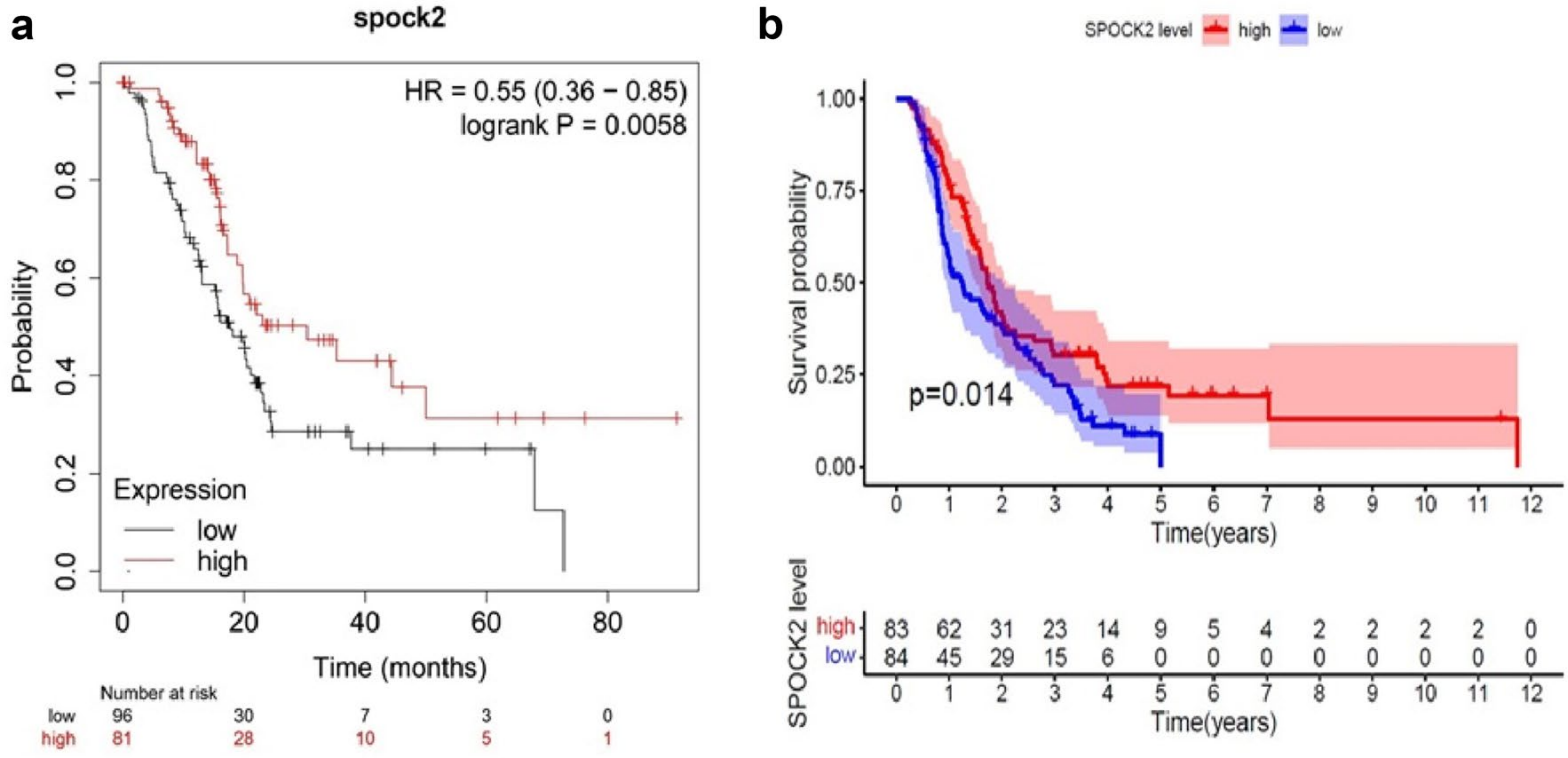
The association between SPOCK2 mRNA expression and the outcome of PDAC patients. **a** KM survival analysis of SPOCK2 in patients with PDAC was performed using KM Plotter. (https://kmplot.com/analysis/). **b** Kaplan–Meier curves were generated for a cohort from the International Cancer Genome Consortium (*ICGC*), with high and low expression levels represented by red and blue lines, respectively

To investigate the association between SPOCK2 mRNA expression and patient outcomes in PDAC, we analyzed the effect of SPOCK2 expression on survival, employing a Kaplan–Meier plotter. Our findings revealed that elevated SPOCK2 mRNA expression was significantly linked to a favorable OS (HR 0.64, 95% CI 0.42–0.96, *P* = 0.031) of patients with PDAC **(**Fig. 8a). To further verify the prognostic value of SPOCK2 in PDAC, a cohort from ICGC with 167 PDAC patients with complete RNA sequencing and prognostic data was investigated. Kaplan–Meier analysis revealed that high SPOCK2 transcriptional levels were significantly correlated with better survival in this cohort as well (Fig. 8b).

## Discussion

SPOCK2 belongs to a group of SPARC proteins, which encompasses various proteins that have been studied in various gastrointestinal malignancies, such as biliary tract cancers (BTC) (Aghamaliyev et al. 2019), PDAC (Vaz et al. 2015), and colon cancer (Zhong et al. 2019). The proteins comprising the SPARC family are engaged in tissue remodeling, wound healing, and cellular migration (Bradshaw 2012). Among these proteins, SPARC is the most studied member in PDAC and has been demonstrated to impact the migration and invasiveness of PDAC cells through a variety of mechanisms (Rossi et al. 2016). Hevin was also reported in PDAC and suggested to serve as tumor-suppressor in PDAC (Esposito et al. 2007). Recently, SPOCK1 was observed to promote PDAC metastasis through EMT (Cui et al. 2022). However, the expression pattern of SPOCK2 and its potential role in PDAC is still unknown. Consequently, the objective of this study was to investigate the biological role of SPOCK2 in PDAC.

An increasing number of investigations focus on SPOCK2 in diverse malignancies, as it was reported to be an attractive tumor marker in cancer in 2008 (Chung et al. 2008). In prostate cancer, in comparison to benign prostate hyperplasia, SPOCK2 was lower in cancer tissue samples (Liu et al. 2019). Similarly, we found that SPOCK2 mRNA level was dramatically lower in PDAC cell lines than in an immortalized normal pancreatic epithelial cell line. Using immunohistochemistry, Ren et al. demonstrated that the protein level of SPOCK2 in type I endometrial cancer was significantly lower than that of the expression of SPOCK2 protein in normal endometrium (Ren et al. 2020).

It is widely recognized that aberrant methylation of promoter CpG islands results in the downregulation of tumor-suppressor genes in the absence of genetic mutations (Jones et al. 1999). In colon cancer, the hypermethylation of SPOCK2 gene was higher in cancer cells than that in the normal mucosal tissue adjacent to the cancer (Sambuudash et al. 2017). As a result, we hypothesized that hypermethylation may underlie the downregulation of SPOCK2 in PDAC. Our investigation revealed a negative correlation between the level of SPOCK2 methylation and its corresponding gene expression. In addition, treatment of PDAC cell lines with 5-aza-dC, led to significant upregulation of SPOCK2 gene and protein expression. These findings suggest that DNA methylation is responsible for the downregulation of SPOCK2 in PDAC.

A recent study indicated that SPOCK2 has the capability to impede the invasion and migration of prostate cancer cells (Liu et al. 2019). As PDAC is one of the aggressive and invasive malignancies, we investigated if SPOCK2 could play a role in PDAC invasiveness as well. We found that the migration ability and proliferation rate of PDAC cells were increased after SPOCK2 knockdown. Furthermore, the silencing of SPOCK2 reduced the gene and protein expression of the epithelial marker ZO-1. These findings provide evidence for the tumor-suppressive role of SPOCK2 in PDAC and suggest the possibility of its downregulation during the EMT. Since SPOCK2 expression was reported to be correlated with overall survival in lung adenocarcinoma (J. Zhao et al. 2020), we performed a prognostic analysis using KM Plotter and ICGC database, and the findings of this study demonstrate a positive correlation between elevated SPOCK2 expression levels and improved OS.

To our knowledge, this study is the first to demonstrate the downregulation of SPOCK2 in PDAC cells, which appears to result from the hypermethylation of SPOCK2. Interestingly, silencing of SPOCK2 leads to stimulation of proliferation and migration of cancer cells, indicating SPOCK2 serves as a tumor suppressor in PDAC. Moreover, higher SPOCK2 expression was correlated with improved OS in patients with PDAC. Collectively, these findings suggest that SPOCK2 might be not only a promising prognostic marker but also an attractive target for PDAC therapy.

## Data Availability

The original data can be provided by the corresponding author upon reasonable request.
